# Clinical outcome and safety study of a newly developed instrumented French-door cervical laminoplasty technique

**DOI:** 10.1007/s10195-016-0440-9

**Published:** 2017-01-25

**Authors:** Luigi Aurelio Nasto, Samiul Muquit, Ana Belen Perez-Romera, Hossein Mehdian

**Affiliations:** The Centre for Spinal Studies and Surgery, Queen’s Medical Centre, Nottingham University Hospitals NHS Trust, Derby Road, Nottingham, NG7 2UH UK

**Keywords:** Cervical myelopathy, Cervical laminoplasty, Motion preservation, Laminoplasty plate

## Abstract

**Background:**

Standard laminectomy for treatment of cervical myelopathy is associated with secondary instability and kyphosis, while laminectomy combined with fusion puts adjacent segments at risk of degeneration. Single- and double-door laminoplasty techniques have been developed to overcome these limitations. More recently, complications related to bone graft dislodgment have fostered development of hardware-augmented laminoplasty techniques. The aim of this study is to review the clinical safety and effectiveness of a newly developed technique of instrumented French-door laminoplasty for treatment of cervical myelopathy.

**Materials and methods:**

A series of 25 consecutive myelopathic patients were treated with a novel instrumented cervical French-door laminoplasty technique, whereby the enlarged posterior arch was held open with maxillofacial plates and screws. Patients had pre- and postoperative assessments with the Neck Disability Index (NDI), Japanese Orthopaedic Association (JOA) Score, Visual Analogue Score and radiographs. Minimum follow-up was 40 months, with regular interval assessments.

**Results:**

There were 18 males with a mean age of 45 years. The mean operative time was 145 min. The average hospital stay was 2.4 days and the mean follow-up was 56.5 months (40–72). All patients reported neurological improvements and there was a 35% improvement in NDI, and JOA score improved by 4.8 points. No postoperative hardware-related complications were noted and only one case of temporary C5 palsy, which completely resolved by the one-year follow-up.

**Conclusions:**

Our data and clinical experience demonstrate that this hardware-augmented laminoplasty technique is safe and effective. We observed no hardware-related complications in our series. The use of readily available maxillofacial titanium miniplates and ease of surgical procedure means that this technique can be easily adopted into clinical practice.

**Level of evidence:**

Level IV.

## Introduction

Degenerative changes in the cervical spine are manifested as disc height loss, facet and uncovertebral joint osteophytes, spondylotic bars, and hypertrophic ligamentum flavum. These changes can lead to central or foraminal stenosis and compromise nerve roots or the spinal cord [1, 2]. The term cervical spondylotic myelopathy (CSM) refers to the presence of a compressive myelopathy due to degenerative changes in the cervical spine. CSM is currently the most frequent cause of cervical myelopathy in the elderly and a significant cause of increased morbidity [3]. Often patients complain of loss of dexterity and weakness in upper limbs; other signs of myelopathy include hyperactive deep tendon reflexes, clonus, pathological reflexes and gait disturbances. CSM is a progressive condition that if left untreated commonly leaves the patient dependent on ambulatory aids and limits activities of daily living [2, 4–6].

The spinal cord in CSM can be compressed by herniated discs, osteophytic bars, and uncovertebral osteophytes. More rare causes of compression of the spinal cord in the cervical spine are ossification of the posterior longitudinal ligament (OPLL), constitutionally narrow spinal canal and ossification of the yellow ligament (OYL) [7]. Several surgical approaches have been developed to address the different causes of compression of the spinal cord. Anterior decompression is very useful in cases where compression of the spinal cord is limited to one or two levels [8, 9]. Moreover, if sagittal alignment of the cervical spine is lost with development of a kyphotic deformity, the anterior approach allows the surgeon to correct the deformity restoring a normal height of the anterior column. In cases with a posterior compression on the spinal cord or multilevel pathology (>2 levels), a posterior approach is preferred [9]. All posterior approaches share the same direct and indirect mechanism of decompression. Removal of the posterior structures of the cervical spine directly relieves compression on the spinal cord, while also allowing posterior shift of the cord away from the impinging anterior structures (indirect decompression). Laminectomy and laminoplasty are the two main techniques available for posterior decompression in the cervical spine.

Cervical laminoplasty was first described by Oyama et al. in the 1970s with the intent of overcoming the limitations of a standard laminectomy procedure. Recurrence of compression on the spinal cord, post-laminectomy instability and kyphosis are well-known complications of laminectomy in the cervical spine [10–12]. Supplementation of laminectomy with a fusion can prevent development of post-laminectomy instability but at the cost of increased rates of adjacent segment degeneration and higher rates of complications related to implant malpositioning [13–15]. Moreover, treatment of CSM in younger patients represents a particular challenge in view of the importance of motion preservation in this particular subgroup of patients. Cervical laminoplasty is a technique designed to allow multiple level posterior decompression of the spinal canal while maintaining spinal alignment and stability through preservation of the posterior elements. Many different laminoplasty techniques have been described but they all share the same principle of achieving decompression of a stenotic spinal canal through the expansion of the posterior arch, the conservation of the posterior elements, and preservation of segmental motion. The techniques can be broadly divided into unilateral hinge, z-laminoplasty, and bilateral hinges (i.e., French-door laminoplasty or two lateral hinges with intact spinous processes). The fundamental aim of all these techniques is to achieve and maintain an expanded spinal canal while preserving motion and alignment of the cervical spine.

French-door laminoplasty was first described by Kurokawa et al. in 1980 to address the limitations of single-door expansile laminoplasty. The midline spinous process split prevented asymmetry of extensor spinal musculature and minimised excessive epidural bleeding [16, 17]. The two lateral hinges opened the cervical canal like a French-door with plastic deformation/greenstick fracture of the troughs and a spacer material (bone graft or bone graft substitute) was placed in between the opened spinous processes to prevent re-closure [18]. Dislodgement of the spacers was a commonly reported complication of the procedure and was described by other authors as well [19]. More recently, several modifications of the original technique have been reported while clinical research has focused on developing instrumentation devices to provide extra stability to the graft and ensure spinal canal patency (i.e., hardware-augmented laminoplasty). Our technique of hardware-augmented French-door laminoplasty consists of fixing the bone graft spacer in between spinous processes to the lamina and lateral masses with maxillofacial titanium miniplates. We have been using this technique for the past 7 years and report our clinical outcomes with this procedure.

## Materials and methods

Following institutional review board approval (as part of service evaluation), the records of 25 consecutive patients surgically treated with our newly developed technique of instrumented French-door laminoplasty at our institution from January 2009 to December 2011 were retrieved from our database. Indication for surgery was the presence of multiple level (≥2 levels) cervical myelopathy of any cause (OPLL, congenital narrow canal, multilevel osteophytic bars) with preserved cervical lordosis (C2–C7 lordosis angle ≥10°). Exclusion criteria included previous surgery in the cervical spine, pre-existing kyphotic deformity, tumors and trauma. All patients had symptoms of cervical myelopathy for at least 8.4 months (range 4–15 months) before surgery.

### Surgical technique

**Fig. 1:**
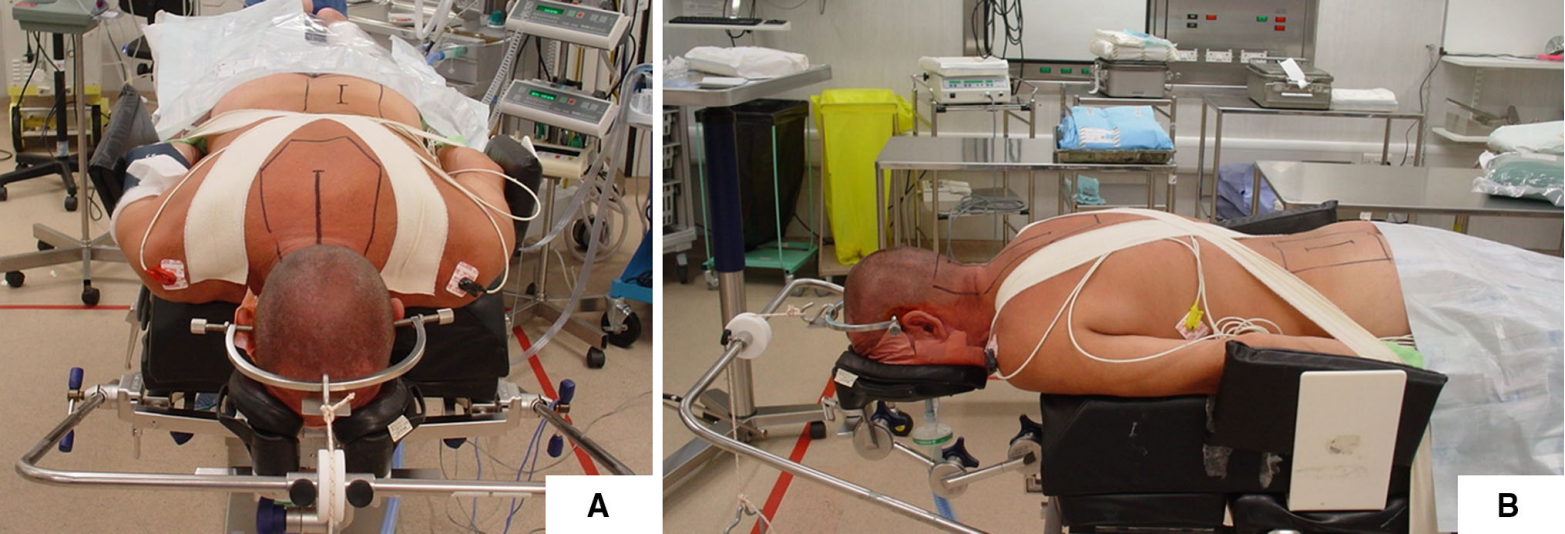
Patient positioning in theatre, front (**a**) and side (**b**) view. Skull traction (5 lb) is applied with Gardner−Well tongs and pressure areas identified and protected

**Fig. 2:**
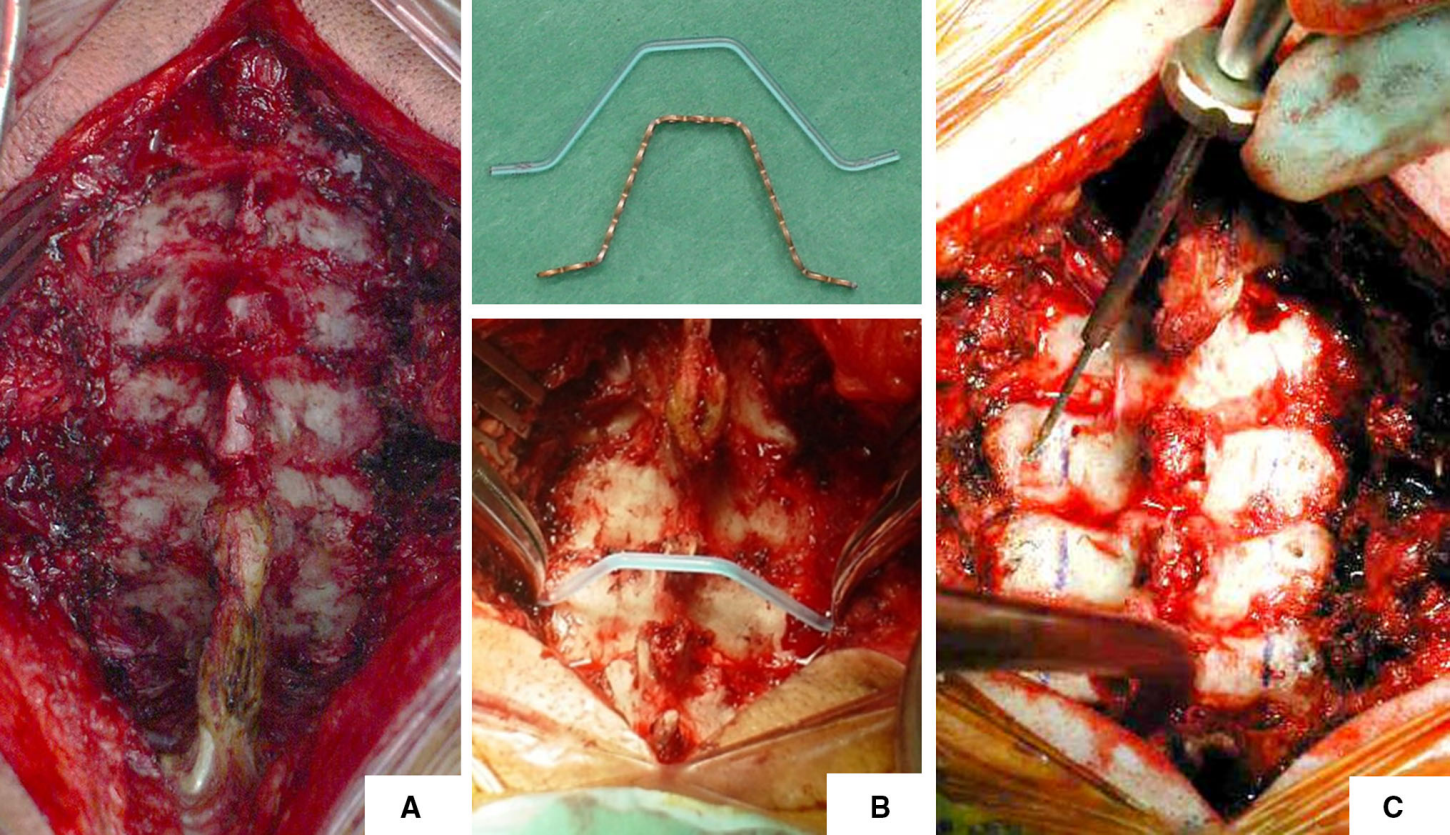
Subperiosteal exposure of lamina and lateral masses with preservation of articular joints. Tips of spinous processes are excised (**a**); a phantom rod is used as template and the miniplate is contoured accordingly (**b**); the screw holes in the lateral masses are prepared before performing the decompression (**c**)

**Fig. 3:**
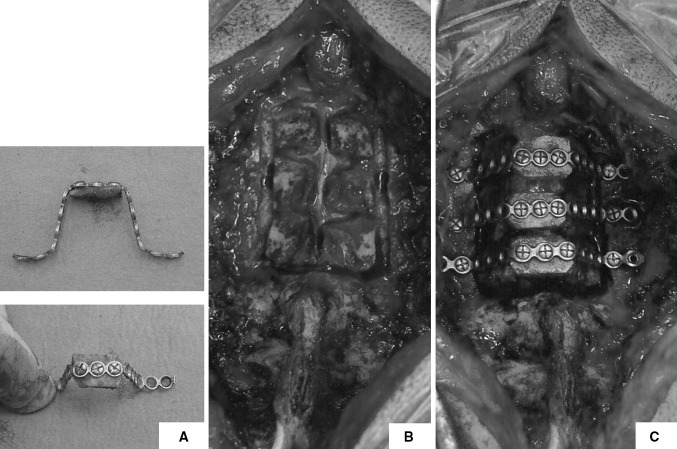
The bone graft is fixed to the contoured miniplate, a 12–15-mm-wide bone graft is used (**a**); bilateral bone troughs are prepared and spinous processes split in the midline (**b**); spinous processes are gently opened on both sides and the miniplates with grafts fitted in place (**c**)

Following general anaesthesia and endotracheal intubation, the patient was positioned prone and in-line cervical traction (5 lbs) with Gardner−Well tongs was applied. Pressure areas were identified and protected and the operating table was positioned in 10°–20° reverse Trendelenburg position to prevent venous congestion (Fig. 1). Surgical sites (posterior cervical spine and left posterior iliac crest) were prepared and draped. Skin and subcutaneous tissues were infiltrated with local anaesthetic and adrenaline solution to minimise bleeding. A midline posterior incision was made and tissues dissected along the median raphe down to the tips of the spinous processes. The posterior elements were exposed by subperiosteal dissection; however, muscular attachments to C2 and C7 were preserved in all patients. Care was taken to expose the lateral borders of the lateral masses while preserving the capsules of the posterior joints. The supraspinous and interspinous ligaments between the planned decompressed area were removed. The bifid spinous processes were excised with a bone cutter. A malleable template was then contoured to sit snugly on posterior elements and a titanium miniplate (Lehbinger, Freiburg, Germany) fashioned into the template’s trapezoidal shape. Care was taken to ensure that the middle portion of the trapezoid was at least 15–20 mm wide. Two screw holes were drilled at planned areas in the lateral masses to facilitate the miniplate anchorage after decompression. A high speed burr (4 mm; Midas Rex Pneumatic Tools, Fort Worth, TX, USA) was used to split the spinous processes in midline. The same high speed burr was used to create unicortical hinges at the lateral gutter (junction of lamina and lateral mass) (Fig. 2). A lamina spreader was then used to gently split the spinous processes and open the French-doors by plastic deformation at the lateral hinges. The midline was expanded 12–18 mm and the underlying ligamentum flavum was excised to visualise the pulsating dura and confirm adequate decompression. Undercutting laminectomy was performed at C2 and C7 level when appropriate to achieve extra decompression. The C7 spinous process with its attached nuchal ligament was left intact in all cases. Unicortical bone graft was harvested from posterior iliac crest and fashioned into as many bone blocks as needed measuring 12–18 × 8–10 mm. The harvested bone graft was fixed in the centre of the 12–15-hole titanium miniplates with two 2.3 × 3−7-mm titanium screws. The final construct (miniplate plus bone graft) was then interposed in between the sagittally split spinous processes and fixed to the lateral masses bilaterally with two 5–11-mm titanium screws. If the lamina is felt to be unstable because of complete fracture at the hinge side it can be fixed to the plate with two extra screws. The same procedure was repeated for each decompressed level (Fig. 3).

All patients were prescribed a soft collar after surgery for pain comfort and they were encouraged to discard the collar by the end of the first postoperative week. All patients underwent active physiotherapy protocol after surgery. Patients were seen in the outpatient clinic at 1, 3, 6 and 12 months after surgery and then at regular 1-year follow-up appointments. Neck Disability Index (NDI), Visual Analog Scale (VAS) for pain, Japanese Orthopaedic Association (JOA) Score, and Nurick’s Grade were assessed in all patients.

### Statistical analysis

Continuous data are presented as mean ± standard deviation, frequency data as counts and percentages. Paired *t* test was used for continuous variables and the chi-squared test for frequency variables. Analysis of variance (ANOVA) for repeated measures was used to assess time differences in JOA and NDI scores across time points, while paired *t* test was used to compare score means between two adjacent time points. A *p* level of ≤0.05 was considered significant. Data were analysed using SPSS statistic software, version 17 (SPSS, Chicago, IL, USA) and Microsoft Office Excel 2007 (Microsoft, Redmond, WA, USA).

## Results

**Fig. 4:**
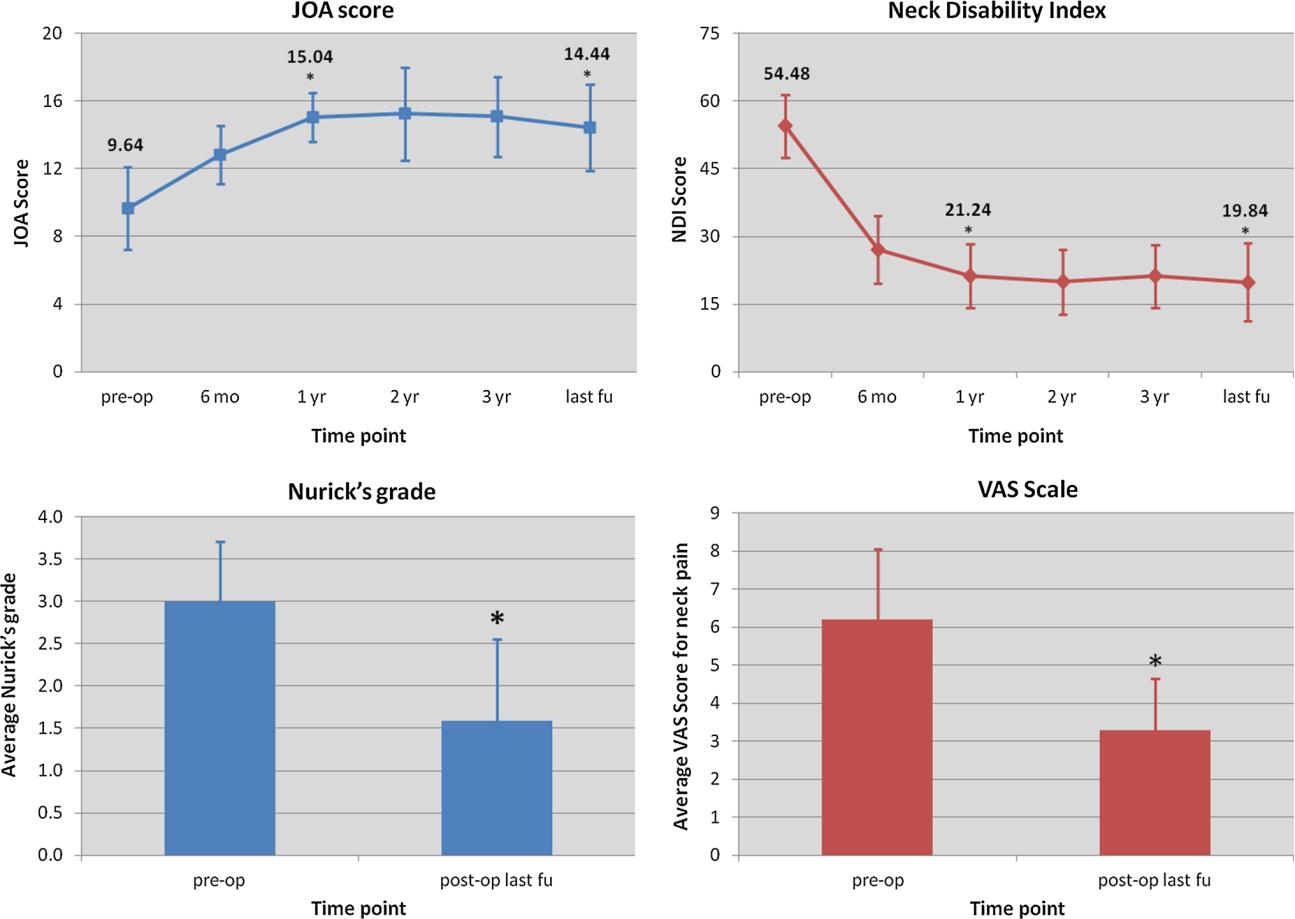
JOA score (*top left panel*), Neck Disability Index (*top right panel*), Nurick’s grade (*bottom left panel*), and VAS scale for axial neck pain (*bottom right panel*). Significant changes (*p* ≤ 0.05) are marked with an *asterisk* in the graphs

The patient demographics (age, underlying pathology, number of treated levels and duration follow-up) are summarised in Table 1. Their clinical features post surgery and at final follow-up with respect to NDI, VAS, JOA score and Nurick’s grade are reported in Fig. 4.

**Table 1:** Study population demographics and clinical characteristics

Variable	
Age (years)	45.3 (38–61)
Follow-up (years)	56.5 (40–72)
Sex (M/F)	18 M, 7 F
BMI (kg/m^2^)	26.3 (22.5–29.0)
Smoking habit (yes/no), *n* (%)	7 yes (27%), 18 no (73%)
Diagnosis Constitutionally narrow canal OPLL Multilevel degenerative disc disease	*N* Levels 7 (28%) 3.2 (3–5) 2 (08%) 3.3 (3–4) 16 (64%) 2.5 (2–5)

**Fig. 5:**
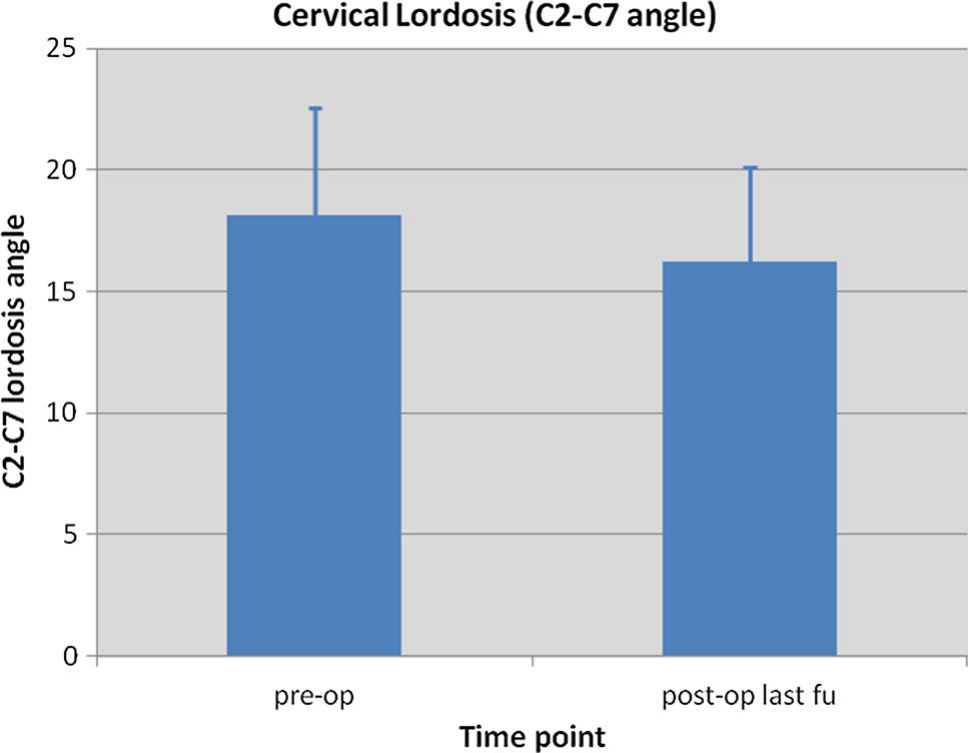
Cervical lordosis (C2–C7 angle) comparison before surgery and at the latest follow-up

The mean age at surgery was 45.3 years (range 38–61) and the mean follow-up was 56.5 months (range 40–72). There were 18 (72%) males in our case series and 7 (27%) smokers. Seven patients (28%) were diagnosed with a constitutionally narrow canal and had an average of 3.2 levels decompressed, 2 patients (8%) in our case series had OPLL, whilst 16 (64%) remaining patients had multilevel degenerative disc disease. The average number of levels decompressed was 3.0 ± 0.8 (Table 1). The JOA score improved from a mean of 9.64 ± 2.44 to a mean of 12.83 ± 1.71 at 6 months after surgery (*p* < 0.001). Continuous improvement of the JOA score was noted up to 2 years after surgery, and then a slight decrease was observed. The JOA score at last follow-up was 14.44 ± 2.56, accounting for a net improvement of 4.8 points [*F*_time point_(3.56, 85.60) = 65.578, *p* < 0.001] (Fig. 4, panel a). The mean pre-operative NDI was 54.48 ± 6.92 and mean NDI at final follow-up was 19.84 ± 8.62, resulting in net improvement at final follow-up of 35% [*F*_time point_(3.33, 80.01) = 137.662, *p* < 0.001]. VAS score was used to assess intensity of neck pain in our patients before and after surgery and was only available in 18 patients (Fig. 4, panel b). The mean preoperative VAS score was 5.30 ± 2.45 and decreased to 3.28 ± 1.37 at final follow-up (*p* < 0.003). All of these 18 patients had some degree of neck pain before surgery and after surgery, although it was significantly improved (Fig. 4, panel c). Average preoperative Nurick’s grade was 3.0 ± 0.71 and improved to 1.58 ± 0.97 at final follow-up (*p* < 0.042) (Fig. 4, panel d). Average cervical kyphosis was 18.12 ± 4.47 degrees before surgery and was slightly decreased at the time of the last follow-up (16.24 ± 3.90, *p* < 0.080) (Fig. 5).

The main intraoperative blood loss was 154 ml (range 85–310) and mean operating time was 145 min (100–245). No intraoperative or early postoperative complications were noted; no hardware complications were encountered during the study period. One patient (4%) had a C5 palsy after surgery which recovered by the 1-year follow-up.

## Discussion

Laminectomy for CSM is associated with 20–43% incidence of post-laminectomy kyphosis/instability and recurrence of canal stenosis (e.g., post-laminectomy membrane) [20, 21]. Addition of a fusion to a standard laminectomy is also associated with loss of segmental motion and increased incidence of adjacent segment degeneration. Laminoplasty was developed as a simple alternative technique to avoid these complications and since its inception has evolved in many different techniques [8, 16, 22].

The main criticism of the single open-door laminoplasty revolves around the asymmetrical expansion of the spinal canal as well as resulting asymmetry of the paraspinal musculature. It has also been associated with increased epidural bleeding and higher incidence of C5 postoperative palsy [17]. Double-door (French-door) laminoplasty was first described by Kurokawa et al. in the 1980s and was developed in order to overcome initial limitations of the single door approach. By splitting spinous processes in the middle, the French-door approach achieves a symmetrical expansion of the spinal canal and less epidural bleeding. Moreover, a lower incidence of postoperative C5 palsy was observed in this technique [23]. However, cases of restenosis because of hinge closure (spring back phenomenon) are a significant concern for both techniques and led to the use of inlay grafts to maintain expanded canal dimensions. To prevent dislodgement of the grafts many different fixation techniques have been developed, including fixation wires and plates [24].

**Fig. 6:**
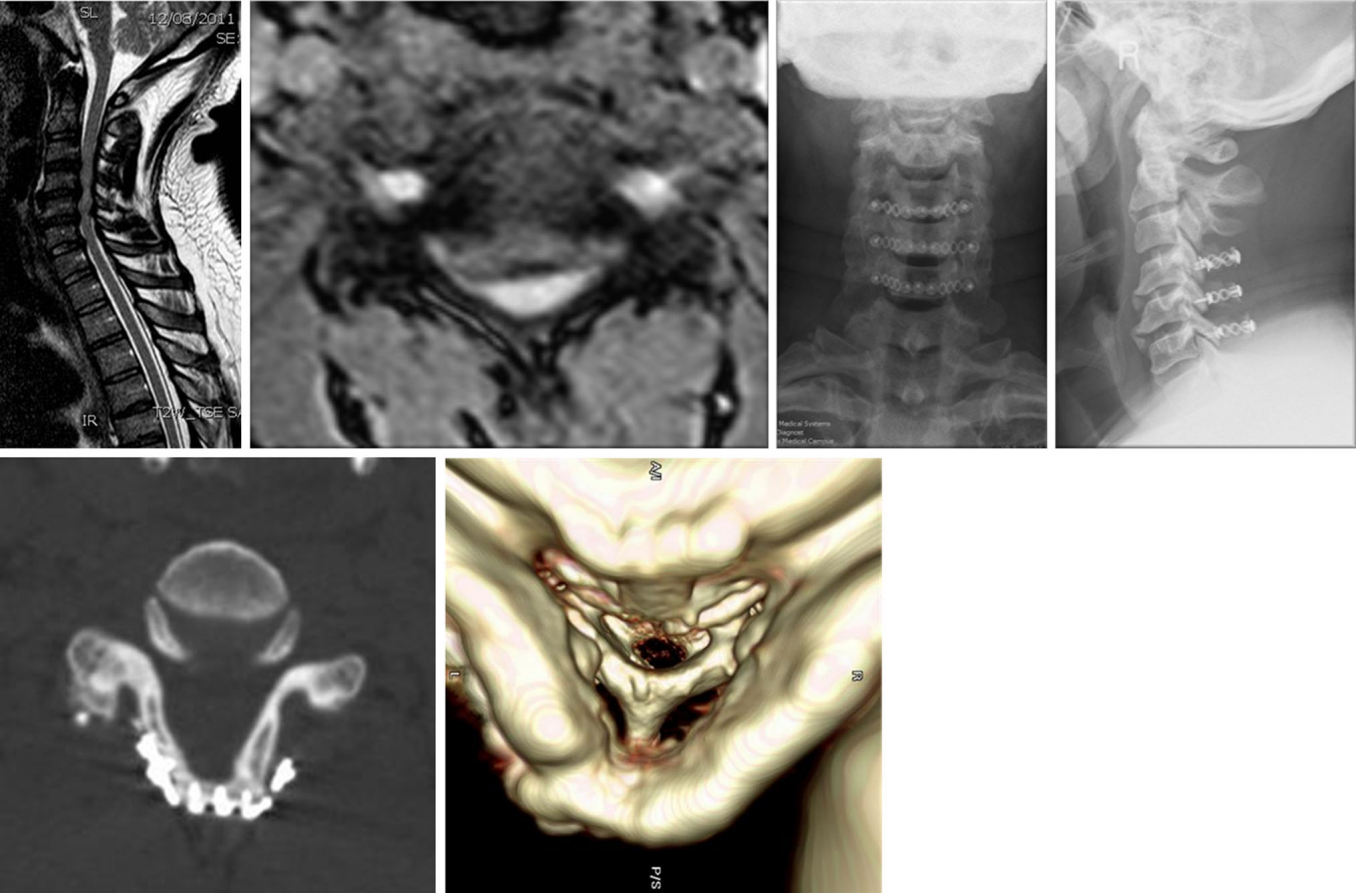
A case example of our modified hardware-augmented French-door laminoplasty technique. Sagittal and coronal views of preoperative MRI are shown with postoperative X-ray of the cervical spine. CT scan analysis at 1 year after the operation shows good decompression of the spinal canal and graft fusion

Our technique involves the use of a single titanium miniplate and screw fixation construct at every level for French-door laminoplasty. Here, we report our clinical experience with this technique and patient outcomes up to 6 years. All our patients had moderate to severe spinal cord compression at multiple levels (≥2). The most common level of compression was from C3 to C6. Our data show a significant improvement in JOA scores (4.8 points) at final follow-up as well as a significant 35% decrease in NDI scores, confirming a successful decompression of the spinal cord. These findings are in keeping with current literature in terms of neurological improvement after surgery [25]. In realising the sagittal split of the spinous processes, we took care to insert a 12–15-mm-wide bone graft at every level. Our choice of graft size is supported by a study by Wang et al. where the authors have shown that a better and significantly bigger cross-sectional area of the spinal canal can be achieved with ≥12-mm-wide bone grafts [26]. Grafts were fixed to the lamina and lateral masses with titanium miniplates and no complications such as graft dislodgment, spring back phenomenon, and hardware failure were noted in our case series. Only one patient had a temporary C5 palsy and this is in keeping with the reported incidence of this complication in double-door laminoplasty (Fig. 6).

Other authors have also reported their experience with hardware-augmented double-door laminoplasty. Goto et al. described a technique involving en-bloc lift of posterior elements with hydroxyapatite spacers in a series of 21 patients [27]. Hydroxyapatite spacers were fixed with two separate plates and screws to the lateral masses. The mean follow-up was 24 months and although the authors reported no incidence of non-union we argue that this is technically more demanding and the results may not be reproducible by other surgeons. In a retrospective study of 54 patients, Chen et al. compared clinical and radiological outcome of patients treated with miniplate and suture-augmented open-door laminoplasty [28]. The authors found that the two techniques were similar in preventing closure of the laminae but the miniplate group had better outcomes in terms of pain and preservation of cervical lordosis. In a similar study, Wang et al. compared two groups of 25 patients who were treated with standard Hirabayashi open-door laminoplasty versus plate-augmented laminoplasty [29]. The authors found similar clinical outcomes in both groups, but less axial pain and a lower incidence of postoperative complications in the hardware-augmented group. Similarly, in our case series we observed a significant decrease of postoperative neck pain VAS scores (5.30 ± 2.45 vs 3.28 ± 1.37, *p* < 0.003) and a slight non-significant decrease of cervical lordosis (18.12 ± 4.47 vs 16.24 ± 3.90, *p* < 0.080).

Our study describes a safe, reproducible and alternative technique for hardware-augmented French-door laminoplasty. Our technique does not require any dedicated spinal implant, but instead uses standard maxillofacial titanium miniplates, which are more readily available in most hospitals. The implantation of titanium miniplates does not preclude the ability to obtain postoperative magnetic resonance imaging (MRI) scans as this is MRI compatible and produces little artefact. The use of our fixation device was shown to be effective in maintaining an adequate decompression of the spinal canal (Fig. 6) as well as significantly reducing postoperative neck pain. One disadvantage of our technique is the use of autologous bone graft for the expansion laminoplasty and the associated donor-site morbidity. Although we do not have direct experience on this, the use of hydroxyapatite spacers with this technique should be carefully considered because of the risk of breakage of the spacer with screwing. The main criticism of our study is the lack of a control group using other fixation techniques (e.g., wiring, suture anchors, or plating). Moreover, no formal biomechanical study has been undertaken to prove long-term stability of the implant. One-year postoperative computed tomography (CT) scan analysis was performed in only seven patients and it confirmed complete fusion of the bone graft in all patients. However, routine CT scan was not part of our postoperative protocol because of radiation exposure considerations. Therefore, a formal study of the bone fusion rate was not undertaken in this study. The achievement of a solid fusion at the graft site is critical to this technique. All our patients were followed up with regular X-rays of the cervical spine and no metalwork complications were noted in any of the patients (e.g., plate failure, breakage, dislodgement) at mean follow-up of 56.5 months. Although this can only be considered as indirect evidence of fusion, it shows that the fixation of the bone graft with miniplates provides a satisfactory stability in the long term. Cervical kyphosis was also preserved in our case series showing no destabilising effect of the implant.
